# Advancements and Applications of Split Technology in CRISPR/Cas12a: Transforming Molecular Diagnostics and Biosensing

**DOI:** 10.3390/bios15090595

**Published:** 2025-09-10

**Authors:** Saikarthik Jayakumar, Srinivasan Vengadassalapathy, Santhosh Venkadassalapathy, Sheela Durairajan, Radha Vijayaraj, Lakshmanan Govindan

**Affiliations:** 1 Department of Maxillofacial Surgery and Diagnostic Sciences, College of Dentistry, Majmaah University, Al Majmaah 11952, Saudi Arabia; s.jaya@mu.edu.sa; 2 Department of Pharmacology, Sri Venkateswaraa Medical College Hospital and Research Institute, Sri Venkateswaraa University, Redhills, Chennai 600067, India; meu@svmedcity.com (S.V.); pharmacology@svmedicity.com (S.D.); 3 Department of Periodontics and Oral Implantology, SRM Dental College, Chennai 600089, India; santhosv@srmist.edu.in; 4 Aquaculture Research Laboratory, Department of Marine Biotechnology, AMET University, Chennai 603112, India; vijayarajr@ametuniv.ac.in; 5 Sri Lakshmi Narayana Institute of Medical Sciences, Affiliated to Bharath Institute of Higher Education and Research, Puducherry 605502, India

**Keywords:** CRISPR/Cas12a, split technology, molecular diagnostics, biosensing, gene editing, nucleic acid detection

## Abstract

The rapid evolution of CRISPR technology has revolutionized molecular biology, and among the various systems, CRISPR/Cas12a stands out for its high specificity and efficient collateral cleavage activity. This review article focuses on the recent advancements and applications of split technology within the CRISPR/Cas12a framework, highlighting its transformative role in molecular diagnostics and biosensing. Split technology innovatively divides functional nucleic acid components into modular segments that are activated by specific targets, significantly enhancing the specificity and sensitivity of biosensors. This design addresses the inherent limitations of traditional sensor systems, enabling the direct detection of ultrashort nucleic acids and improved discrimination of single-nucleotide variants, thereby facilitating the simultaneous detection of multiple biomolecules. The versatility of split-enabled biosensors extends beyond genetic testing, making them valuable tools in diagnostics, therapeutics, and environmental science. Despite challenges such as crRNA degradation and reassembly kinetics, ongoing research and engineering solutions continue to enhance the stability and performance of these systems. This review synthesizes the foundational principles, recent advancements, and potential applications of split technology while also identifying challenges and opportunities for future exploration. Ultimately, our insights provide a comprehensive resource to leverage the full potential of CRISPR/Cas12a-based split technology in advancing biosensing methodologies and clinical applications.

## 1. Introduction

The rapid advancement of CRISPR technology has established itself as a transformative force in molecular biology and biotechnology, providing researchers and clinicians with powerful tools for precise gene editing and manipulation [1]. Among the various CRISPR systems, CRISPR/Cas12a has emerged as a particularly valuable platform due to its unique properties, including its ability to recognize a wide range of nucleic acid targets with high specificity and its efficient collateral cleavage activity. Originally derived from microbial immune systems, the CRISPR/Cas12a system has swiftly transitioned to become a key player in various applications, including molecular diagnostics, gene therapy, and biosensing. Its programmability and versatility enable the development of sensitive detection methods for diverse genetic materials, with significant implications for clinical diagnostics, environmental monitoring, and agricultural biotechnology [1,2]. A notable advancement within the framework of CRISPR/Cas12a is the emergence of split technology, which provides an innovative approach to enhance the specificity and functionality of biosensors [3]. Unlike traditional designs that employ single-component crRNAs, split technology involves dividing functional nucleic acid components, primarily the CRISPR RNA (crRNA) or activator strands, into two or more separate segments [3]. These segments remain inactive until they are assembled or activated by the presence of a specific target or analyte [4]. This modular design enables a more sophisticated detection mechanism, significantly reducing off-target interactions and background noise [5]. By allowing a higher degree of programmability, split technology represents a promising avenue for developing next-generation biosensors with improved performance and reliability [4].

The introduction of split technology into CRISPR/Cas12a-based sensors is motivated by the need to address several inherent limitations associated with traditional sensor designs. Conventional CRISPR/Cas12a systems often face challenges in directly detecting short nucleic acid targets and accurately distinguishing point mutations and multiple target sequences within a single assay [5,6]. By strategically engineering split sites within the crRNA, researchers can create biosensors that respond with remarkable specificity and sensitivity to target analytes [1,4,6]. This capability allows for direct detection of ultrashort nucleic acids, enhances the discrimination of single-nucleotide variants, and facilitates the design of versatile sensor systems capable of simultaneously detecting multiple biomolecules [1].

The practical applications of split technology extend beyond genetic testing, showcasing its adaptability in detecting a myriad of biological targets, including DNA, RNA, proteins, and small molecules [6]. This versatility renders split technology a transformative tool in various fields, including diagnostics, therapeutics, and environmental science [4,6]. For instance, split-enabled biosensors facilitate the direct detection of microRNAs without the need for reverse transcription, which streamlines assay procedures while significantly enhancing sensitivity [6]. Furthermore, the capability to distinguish single-base mutations is crucial in precision medicine, especially in oncology, where the timely detection of mutations directly impacts patient treatment and outcomes [7]. Despite the remarkable advantages offered by split technology, its implementation is not without challenges. Issues such as crRNA degradation, complexities in designing split elements, suboptimal reassembly kinetics, and potential limitations in detecting long-stranded RNA present significant obstacles [1,4,6]. Addressing these challenges necessitates innovative engineering solutions, including the development of short DNA activators, the application of nucleic acid nanotechnology, and the strategic placement of split sites to improve system stability and compatibility with a wide range of molecular targets [6]. The continuous evolution of split technology within CRISPR/Cas12a systems marks a significant advancement in biosensing capabilities, propelling ongoing research aimed at enhancing its sensitivity, stability, and overall performance [7,8]. As the field progresses, a deeper understanding of the underlying mechanisms, combined with technological advancements, promises to unlock a new wave of applications that can significantly impact diagnostics and therapeutic strategies [1,4,6,7].

This review article aims to provide an in-depth overview of the status and future directions of split technology within CRISPR/Cas12a systems. We will explore the fundamental principles underlying this innovative approach, highlight recent advancements, discuss potential applications across various domains, and identify existing challenges and opportunities for future research. By synthesizing these findings, this review will serve as a valuable resource for researchers and practitioners aiming to harness the full potential of CRISPR/Cas12a split technology in advancing molecular diagnostics and biosensing applications. Ultimately, understanding this rapidly evolving field offers the possibility to drive not only scientific discovery but also the implementation of transformative diagnostic tools and therapeutic innovations. We reviewed peer-reviewed articles and relevant patents published within the last five years. The selection criteria focused on studies discussing the principles, design, and applications of split technology in biosensing. We synthesized findings related to experimental methodologies and performance outcomes, providing a comprehensive overview of the innovations and challenges in the field. This structured approach aims to inform researchers of current trends and guide future investigations in CRISPR/Cas12a applications.

## 2. Principles and Mechanisms of Split Technology in CRISPR/Cas12a Systems

In this subsection, we explore the principles and mechanisms that underpin split technology within CRISPR/Cas12a systems. By dividing crucial molecular components into fragments that reassemble in the presence of specific targets, these systems offer enhanced control over activation, specificity, and flexibility, revolutionizing biosensing applications and genetic engineering strategies.

### 2.1. Fundamental Architecture of Split Technology in CRISPR/Cas12a Systems

Split technology in CRISPR/Cas12a-based systems revolves around artificially dividing critical nucleic acid elements—such as crRNAs or activator strands—into two or more fragments that, when combined, are inactive but reconstitute function upon target-induced assembly or in response to specific triggers [9,10]. Canonically, Cas12a operates with a single crRNA composed of a 5′-handle (scaffold) and a 3′-spacer region that together guide the enzyme to a complementary nucleic acid target, triggering target-specific (cis) cleavage as well as robust collateral (trans) cleavage of single-stranded DNA reporters [9,11]. In split designs, this highly orchestrated interaction is conditionally controlled, allowing precise regulation over when and how the sensor is activated.

### 2.2. Types and Formats of Split Elements: crRNA and Activator Splitting

A primary approach involves separating the crRNA into distinct moieties: the scaffold (or repeat) and the spacer [11]. The scaffold is invariant and responsible for Cas12a binding and structural stability, while the spacer provides programmable target specificity [11]. When these elements are supplied separately, only their successful co-localization and hybridization—often mediated or facilitated by the target nucleic acid itself—restore the canonical crRNA structure, thus reconstituting the Cas12a activation potential [12]. In addition, the crRNA can be further engineered: “splice-at-will” strategies allow for the crRNA to be split at nearly any point within the spacer, generating truncated crRNA (tcrRNA) that can remain inactive unless an auxiliary (often target-derived) RNA or a synthetic activator bridges the gap, completing the functional guide RNA [10]. Beyond crRNA, split activator designs have emerged as powerful alternatives, especially for target-induced assembly [9]. In this format, the traditional single-stranded DNA (ssDNA) or RNA activator sequence, essential for initiating collateral cleavage, can be divided into two or more fragments [9]. These fragments may reassemble in response to a specific nucleic acid analyte, chemical reaction, or hybridization event, generating the active form that triggers Cas12a’s trans-cleavage activity [13]. These modular splits greatly enhance detection flexibility and enable conditional, “AND-gate” style activation pathways for integrated biosensing and logic functions [9].

### 2.3. Mechanisms of Target-Induced Assembly and Activation

Split systems utilize proximity effects and molecular recognition to convert the presence of an analyte into sensor activation [13]. For split crRNA systems, one critical mechanism is target-assisted assembly, whereby the presence of a complementary DNA or RNA causes the two crRNA fragments (scaffold and spacer) to associate, correctly orienting them for Cas12a binding and activation [11]. The efficiency and specificity of this reassembly depend on carefully optimized hybridization stability, fragment lengths, and sequence complementarity [10]. In “splice-at-will” designs, truncated crRNAs with a missing spacer region are complemented by exogenous RNAs, such as ultrashort miRNAs or application-specific targets, forming a complete crRNA sequence that prompts robust Cas12a activation [10]. For split activator systems, either a chemical reaction (e.g., hydrazone bond formation), DNA strand displacement, enzymatic cleavage, or a simple target hybridization event can unite the split parts into a full-length activator [13]. Hydrazone chemistry offers a striking example: two activator fragments, modified with hydrazine and aldehyde groups, respectively, remain inert until brought into proximity—often by a target-induced strand displacement—at which point they form a hydrazone bond, reconstructing the canonical activator and thus initiating the Cas12a response [13]. This chemical proximity effect simultaneously accelerates assembly and enriches specificity, as even single-nucleotide mismatches within the target can strongly inhibit activator formation and subsequent signaling [13].

### 2.4. Modulation of Sensor Activity and Specificity

A key advantage of split technology is its capacity for highly tunable sensor responses [9]. The assembly parameters—such as redundancy (extra nucleotides at the split junction), split site, fragment concentration, and hybridization domains—can be meticulously programmed to achieve fine-grained control over Cas12a activity [9]. For example, introducing mismatches, short gaps, or overhangs at the junction between tcrRNA and its complementary RNA can dramatically modulate, or even abrogate, Cas12a trans-cleavage, affording exquisite sequence discrimination [10]. Split activator systems can leverage redundancy and position-dependent structures to provide an adjustable activation threshold, supporting complex logic operations and facilitating the integration of upstream molecular networks, such as DNAzymes or aptamer assemblies [9]. The specificity imparted by split designs is frequently superior to that of conventional non-split systems, as only the correct assembly of all split components—often relying on perfect sequence complementarity—restores functional activation; thus, off-target or background cleavage is inherently minimized [11,13]. Likewise, by requiring target-induced reassembly, split technology can distinguish between highly similar sequences, such as mature and precursor miRNAs, or discriminate single-nucleotide variants crucial for precise diagnostics [10].

### 2.5. Broad Applicability: Sensing, Logic Operations, and Multiplexing

The principles established by split technology enable much more than conventional biosensing [14]. Beyond straightforward detection, split CRISPR/Cas12a components function as molecular logic gates (“AND,” “OR,” “NOT,” “NAND”), where sensor activation occurs only when precise molecular-input conditions are satisfied, such as the simultaneous presence of two different nucleic acids or enzymes [9]. Modular platforms utilizing these mechanisms have demonstrated simultaneous detection of different SNPs, enzyme activities, and integration within multilayered molecular reaction networks [9]. This approach supports seamless multiplexing, where each unique pair of split fragments may be selectively activated by a specific target, reducing cross-talk and enhancing assay scalability [11].

### 2.6. Structural and Biochemical Insights Supporting Split Functionality

Detailed structural and biochemical studies reveal that the split RNAs or activators, upon correct assembly, closely mimic the native conformation required for high-affinity Cas12a binding and activation [10]. For split crRNAs, the hybridized scaffold-spacer mimics the full-length crRNA: experimental evidence shows near-equivalent rates of cis- and trans-cleavage compared to the wild-type system, provided the split is optimally designed, and hybridization is efficient [10,11]. Preassembly conditions, fragment ratios, and sequence optimization are crucial for achieving native-like activity and strong signal outputs [10].

In summary, the underlying principles of split technology in CRISPR/Cas12a are grounded in molecular engineering that enforces strict analyte dependence for functional assembly, capitalizing on proximity effects, hybridization fidelity, and controlled chemical reactions to translate target presence into robust and specific sensor responses [11,13]. These strategies offer versatile, modular, and highly programmable platforms for biosensing, diagnostics, and bio-computational logic, positioning split CRISPR/Cas12a systems among the most adaptable emerging technologies in synthetic biology and molecular diagnostics [9,10].

## 3. Engineering Strategies and Variants of Split Systems

This subsection highlights innovative engineering strategies for split CRISPR/Cas12a systems, focusing on crRNA bifurcation, split activators, and advanced designs, such as “splice-at-will” and spatially blocked systems. These approaches enhance programmability, specificity, and functionality, enabling highly sensitive and versatile molecular diagnostics.

### 3.1. Engineering Strategies for Split crRNA and Activators

The engineering of split systems in CRISPR/Cas12a-based sensors is fundamentally centered on increasing the programmability, specificity, and functional versatility of CRISPR diagnostics by dividing critical nucleic acid components into multiple fragments that require conditional assembly for activity [10]. The most widely employed strategy involves splitting the crRNA, which guides Cas12a to its target, or the activator sequence, into discrete segments that alone are inactive but regain functionality when brought together by target-specific interactions or external triggers [11]. This modular assembly leverages advances in nucleic acid nanotechnology and chemical biology to finely regulate Cas12a activation in response to diverse molecular cues [9,14].

### 3.2. Split crRNA: Scaffold and Spacer Segmentation

A prominent and foundational approach is the bifurcation of the Cas12a crRNA into its structural domains: the handle-like repeat region (scaffold) and the variable target-recognition region (spacer) [11]. In this split crRNA strategy, the 20-nucleotide (nt) scaffold RNA and the 20-nt spacer RNA are provided separately [15], allowing for flexible reconstitution of the active crRNA only when both segments are present and correctly hybridized [11]. This design is highly programmable, as different spacers can be matched with a universal scaffold to target multiple sequences, enabling straightforward multiplexing and rapid adaptation to new targets [11]. Structural studies confirm that, upon hybridization, the scaffold and spacer functionally mimic the native crRNA, restoring both target binding and trans-cleavage activities of Cas12a with comparable efficiency to wild-type systems when optimally designed [11].

### 3.3. “Splice-at-Will” Strategies and Flexible Spacer Truncation

Innovative “splice-at-will” engineering further extends the split concept by allowing the crRNA to be cleaved at almost any site within the spacer region [10]. This results in a truncated crRNA (tcrRNA) that, by itself, is functionally inert but which regains activity upon hybridization with ultrashort target RNAs (as short as 6–8 nucleotides), thereby forming a functional crRNA capable of activating Cas12a [10]. This strategy is especially valuable for the direct detection of ultrashort microRNAs (miRNAs), enabling acceptable discrimination of single-nucleotide differences through precise control of the reassembly site and RNA complementarity [10]. The flexible truncation site design also allows tailoring the system for sequence specificity, sensitivity, and adaptability to many types of RNA targets [10].

### 3.4. Split Activator and Chimeric Activator Designs

Split activator engineering involves dividing the canonical activator—typically a single-stranded DNA (ssDNA) or hybrid DNA-RNA strand—into two or more separate fragments [16]. Each fragment alone is incapable of activating Cas12a, but reconstitution through target-induced hybridization, chemical ligation, or logic-gate-like assembly restores the full activator, thereby triggering robust trans-cleavage activity [16]. For example, the split nucleic acid-activated Cas12a (SNA-Cas12a) system exploits this principle for direct miRNA detection, where a short single-stranded DNA (ssDNA) is added to the crRNA spacer to facilitate direct RNA recognition and enhancer strand binding [16]. Chimeric DNA-RNA hybrid activators further expand versatility by utilizing fragments of both nucleic acids, which can be conditionally assembled by the target [16]. Hydrazone chemistry-mediated split systems exemplify a powerful chemically inducible strategy [13]. Here, two split activator strands are designed to have reactive chemical groups (such as hydrazine and aldehyde) at their termini. Only upon target-induced proximity, often through hybridization or a strand displacement reaction, are these fragments chemically ligated into a full-length activator, thus providing both spatial and chemical specificity while simultaneously enhancing selectivity for single-nucleotide mismatches [13].

### 3.5. Spatially Blocked Split Systems for Small Molecule Detection

Recent variants introduce spatial constraint-based control by engineering split crRNA systems that can be transiently blocked through the introduction of macromolecules or chemical modifications at the 3′ end of the scaffold [14]. In spatially blocked split CRISPR-Cas12a (SBS-Cas) systems, the 3′ end is chemically modified, such as by conjugating small molecules like biotin or acrylamide [14]. Macromolecules (e.g., streptavidin, antibodies, DNA strands) bind to these modifications, exerting spatial hindrance that occludes proper assembly of the scaffold with Cas12a and renders the system inactive [14]. Upon introduction of a free small molecule analyte (such as biotin, m6A, or glutathione), competitive binding displaces the blocking agent, releasing the scaffold to assemble with Cas12a and thus activating the biosensor [14]. This modular design offers low background signals, enhanced adaptability to various small molecule targets, and ultra-sensitive quantitative detection [14,17].

### 3.6. Key Variants of Split System Implementations

#### 3.6.1. Scaffold-Spacer Split crRNA (SCas12a)

The canonical 40-nucleotide (nt) CRISPR RNA (crRNA) is split into two components: a universal 20-nt scaffold RNA that provides structural stability and a programmable 20-nt spacer RNA that facilitates target recognition [16]. This approach offers straightforward assembly, a versatile scaffold applicable across various uses, rapid adaptability for targeting new sequences, the capability for multiplexed detection of multiple targets simultaneously, and the ability to differentiate between mature microRNAs (miRNAs) and their precursor forms. It is utilized for the direct detection of miRNAs, precise discrimination of point mutations, development of multiplexed assays, and quantification of cancer biomarkers in clinical samples [16].

#### 3.6.2. Splice-at-Will crRNA

The crRNA undergoes truncation at various sites within the spacer sequence, with activation contingent upon its hybridization with target ultrashort RNAs [10]. This method facilitates the direct and sensitive detection of ultrashort RNAs, ranging from 6 to 8 nucleotides, allowing for precise single-nucleotide discrimination and offering significant design flexibility for various RNA species. It is beneficial for the quantification of microRNAs and ultrashort RNAs, as well as for distinguishing single-base variants [10].

#### 3.6.3. Split Activator Approaches

Single-stranded DNA (ssDNA) or chimeric DNA/RNA activators necessitate target-induced reassembly, often leveraging competitive or logic-gate reactions to enhance functionality [16]. This approach offers improved specificity and programmability alongside the potential to integrate AND/OR logic functions within biosensor platforms. The method is applied for direct detection of microRNAs, multi-target sensing, and executing molecular logic computation [16].

#### 3.6.4. Chemically Mediated Assembly

Utilizing in situ chemical ligation techniques, such as hydrazone bonding, allows for the assembly of split activators exclusively in the presence of target molecules [13]. This method enhances specificity through both sequence and chemical selectivity, proving effective for discriminating single-base mismatches. Potential applications include the detection of bacteria and the analysis of rare genetic variants [13].

#### 3.6.5. Spatially Blocked Split Systems (SBS-Cas)

The 3′-end of the scaffold strand is modified with small molecules, while macromolecules mask activity through spatial hindrance. These macromolecules can be released by competitive binding with the analyte [14]. This approach offers a low background, modularity, and high adaptability for small molecule biosensing, making it suitable for intracellular imaging. It has applications in the detection of biotin, acrylamide, reduced glutathione, m6A, puromycin, folic acid, and more, as well as real-time intracellular biomolecule imaging [14].

#### 3.6.6. 1nt DNA-Extended Scaffold Strategy

A single deoxyribonucleotide is appended to the 3′ end of the RNA scaffold, enabling broader chemical conjugation and small molecule coupling [14]. This approach reduces the complexity of modification, expands the detection range, and enhances practicality for various targets. It enables the detection of diverse analytes, including small molecules, antigens, and drugs, through simplified nucleic acid engineering [14].

### 3.7. Integration of Split Strategies with Sensing Platforms

**Figure 1:**
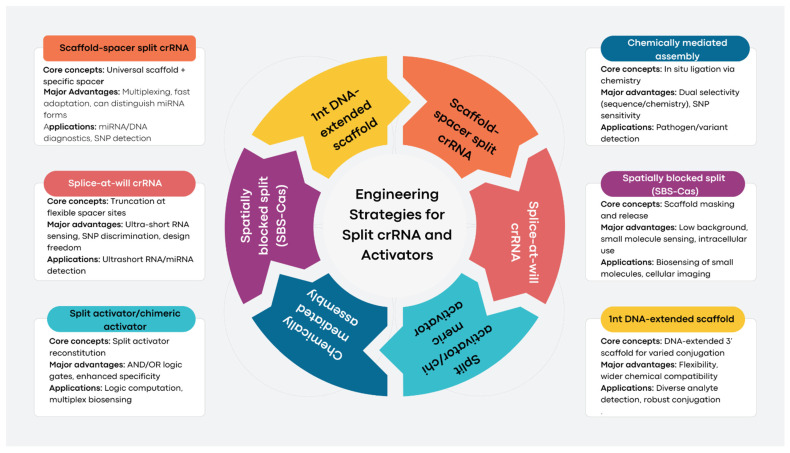
Schematic illustration of the wild-type Cas12a RNP [11]. b Schematic illustration of the split Cas12a RNP. Schematic illustration of c. Splice-at-will’ Cas12a crRNA engineering [10], d. split activators [16].

Advancements in split engineering are increasingly integrated with various detection modalities (fluorescence, lateral flow, electrochemical) and coupled with amplification techniques (e.g., RPA, isothermal, or zero-amplification formats) to produce robust, field-deployable diagnostics (Figure 1) [11]. The modularity and orthogonality of split designs further enable scalable multiplexing and parallel detection of multiple targets with reduced cross-talk [18]. The evolution of split system engineering in CRISPR/Cas12a-based sensors has introduced a spectrum of variants—ranging from canonical scaffold-spacer splits to chemically mediated and spatially blocked systems—that collectively offer unprecedented programmability, specificity, and adaptability for both nucleic acid and small molecule biosensing [11,14]. By leveraging these strategies, researchers can design next-generation molecular diagnostics with broad applications in clinical, environmental, food safety, and bioimaging fields [2,14].

### 3.8. Split Cas12a vs. the Complete Cas12a System

The CRISPR-Cas12a system, a powerful RNA-guided endonuclease derived initially from bacterial immune systems, is utilized widely for nucleic acid detection and genome editing. Its unique ability to specifically target and cleave DNA, along with a mechanism that indiscriminately degrades non-targeted single-stranded DNA (ssDNA), makes it particularly effective for rapid diagnostics [19,20]. There are two primary configurations of the Cas12a system: the complete Cas12a system and the engineered split Cas12a system, each offering distinct advantages and disadvantages in terms of performance metrics such as sensitivity, specificity, detection time, and scalability [21,22,23]. The complete Cas12a system operates as a full-length enzyme guided by CRISPR RNA, forming a 20-nucleotide R-loop to recognize and cleave target DNA. Its trans-cleavage activity enhances sensitivity by facilitating indiscriminate cleavage of non-specific ssDNA, aiding in signal amplification. This system is notable for its rapid kinetics and efficiency, making it suitable for genome editing and swift nucleic acid detection [21,22,23]. Conversely, the split Cas12a system is designed with the Cas12a protein divided into inactive fragments that only become active when specific conditions, such as the presence of target nucleic acid, induce their reassembly. This mechanism significantly reduces background activity and enhances specificity, minimizing false positives. However, the added kinetic steps involved in reassembly can lead to slight trade-offs in detection speed. Still, when paired with amplification techniques, split Cas12a systems exhibit high sensitivity, making them ideal for applications requiring precise discrimination, such as identifying single-nucleotide polymorphisms (SNPs). In terms of sensitivity and detection capabilities, complete Cas12a systems excel, detecting targets in the picomolar range and achieving even greater sensitivity when coupled with amplification techniques like RT-LAMP. They have demonstrated the ability to identify low viral loads in clinical settings [21,22,23]. Similarly, split Cas12a systems also achieve high sensitivity, particularly when combined with amplification strategies, benefiting from their controlled activation for precise applications. Ultimately, the choice between a complete or split Cas12a system hinges on the specific requirements of the application, balancing performance characteristics to optimize outcomes in diagnostics and genome editing [19,20,21,22,23]. The following table provides a detailed comparison of these two systems [22] (Table 1).

## 4. Practical Application of Split Cas12a Technology in Clinical Samples

**Figure 2:**
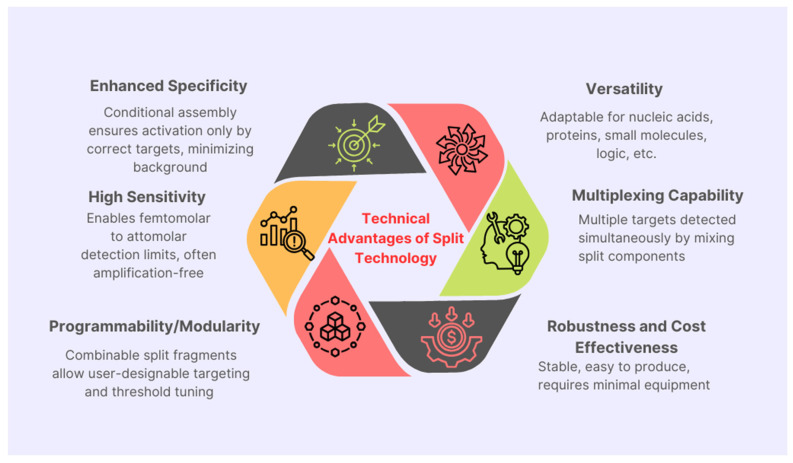
Overview of key advantages offered by split CRISPR/Cas12a technology in precise genome editing applications and enhanced target specificity.

The application of split Cas12a technology in clinical samples, particularly blood and saliva, addresses critical challenges in diagnostic accuracy, operational simplicity, and scalability [29]. These biological fluids offer non-invasive collection and rich biomarker potential, but their complex matrices present significant hurdles for molecular assays. Split Cas12a, with its enhanced specificity and controlled activation, is uniquely positioned to overcome these challenges [30,31,32] (Figure 2).

### 4.1. Sample Handling and Preprocessing in Blood and Saliva

Adequate sample preparation is paramount for the successful deployment of Cas12a-based assays in clinical settings [33]. For saliva samples, which are non-invasive and easily collected, protocols often focus on minimal preprocessing to maintain user-friendliness and speed [33]. Chemical treatments coupled with heat inactivation effectively stabilize viral RNA, enabling direct detection without the need for RNA extraction, which is typically a bottleneck in traditional molecular diagnostics [29,33]. This extraction-free approach not only simplifies the workflow but also maintains high sensitivity and specificity, with reported agreements exceeding 97% compared to traditional RNA extraction methods for SARS-CoV-2 detection [29,30]. The detection of high viral loads in saliva has also been observed, further validating its utility as a diagnostic specimen [29,30]. Blood samples present greater complexity due to the presence of various cellular components, proteins, and inhibitors like hemoglobin, which can interfere with enzymatic reactions. Therefore, optimized chemical treatments and controlled heat inactivation are crucial to balance RNA preservation and viral inactivation [30,34]. These pre-treatment steps enable direct assay application while mitigating false positives arising from matrix effects. Integrated microfluidic systems are particularly beneficial here, streamlining the preparation steps and leading to efficient assay performance [28,34].

### 4.2. Addressing Matrix Effects, Inhibitors, and Background Noise

Clinical samples like blood and saliva contain a variety of biomolecules, enzymes, and endogenous inhibitors that can significantly impair the performance of nucleic acid detection assays [35]. These inhibitors can degrade target nucleic acids or impede the activity of enzymes crucial for Cas12a’s collateral cleavage function, potentially reducing sensitivity and specificity [35]. The intrinsic design of split Cas12a offers a robust solution to these issues [32]. By requiring the reassembly of inactive fragments for activation, the system inherently reduces background cleavage and minimizes false positives, thereby improving the signal-to-noise ratio in complex biological matrices [32]. This conditional activity means that the collateral function is less likely to be triggered by non-specific interactions or endogenous components within the sample [24,32]. Furthermore, strategies such as optimized buffer compositions and the inclusion of specific cofactors, like manganese ions, significantly enhance the sensitivity and accelerate the collateral cleavage kinetics of Cas12a, compensating for potential delays due to fragment reassembly and maintaining rapid detection times even in the presence of inhibitors [24]. This synergistic approach allows for the sensitive and specific detection of low-copy nucleic acids directly from minimally processed saliva and blood [24].

### 4.3. Integration with Point-of-Care (POC) Devices

The development of point-of-care (POC) diagnostics is a primary focus in clinical applications to enable rapid, decentralized testing [31,36]. Split Cas12a assays are highly amenable to integration into POC devices due to their potential for simplified workflows and robust performance in minimally processed samples. Key protocols for integrating split Cas12a assays into POC devices for blood and saliva include Minimal Fluid Handling: Designs favor devices that require minimal manual steps, reducing the chance of human error and cross-contamination [31,36]. Single-tube or cartridge-based systems are ideal. Isothermal Reaction Conditions: Many Cas12a applications are compatible with isothermal amplification methods (e.g., RT-LAMP, RAA), which eliminate the need for complex thermocycling equipment, simplifying device design and power requirements [32,33]. Portable Readout Systems: Integration with smartphone-based fluorescence readouts or lateral-flow assays makes detection accessible and user-friendly, supporting deployment in resource-limited settings [29,37]. For instance, the CASSPIT system uses CRISPR-Cas13a-based SARS-CoV-2 detection with a lateral-flow assay readout, showing 98% agreement with RT-qPCR data for clinical samples, and integrates with a smartphone application for user-friendly results [29]. Reagent Stability: Ensuring the long-term stability of split Cas12a components, particularly their ability to reassemble efficiently, is critical for POC applications. Optimized buffer systems and lyophilized reagents contribute to extended shelf life [32,33]. These integrations aim to make highly accurate molecular diagnostics available outside traditional laboratory settings, which is crucial for disease management and outbreak control [31].

### 4.4. Automation and Scalability for High-Volume Testing

The demand for widespread and rapid testing, particularly during public health crises like the COVID-19 pandemic, highlights the need for scalable and high-throughput diagnostic solutions [33,38]. Split Cas12a assays, like their complete counterparts, are increasingly being integrated into automated platforms to address this need [28]. Integrated microfluidic systems, such as centrifugal microfluidic platforms, can automate the entire diagnostic process from sample preparation to detection [28]. The CASMEAN system, for example, integrates recombinase-aided amplification (RAA) with the Cas12a system to rapidly detect nucleic acids within 1.5 h, making it suitable for high-volume testing in clinical laboratories [28,39]. Automation significantly reduces hands-on time and operational complexity, allowing for the processing of numerous samples concurrently [39]. This is particularly important for high-volume blood and saliva testing, where the rapid turnaround of results can significantly impact public health efforts [38]. The non-invasive nature of saliva collection makes it an attractive specimen for large-scale screening and population-based surveillance [38,39]. Pooled sample testing, where multiple saliva samples are combined and tested together, can further increase testing capacity, especially in low-prevalence settings [38]. However, it requires careful consideration of sensitivity reduction for weakly positive samples. Split Cas12a’s high specificity makes it well-suited for such pooling strategies by minimizing false positives that could arise from non-specific reactions in pooled complex matrices.

### 4.5. Multiplexing and Enhanced Specificity in Clinical Diagnostics

The enhanced specificity of split Cas12a technology, stemming from its conditional activation mechanism, makes it particularly advantageous for multiplexed detection in clinical samples [24]. Multiplexing allows for the simultaneous detection of multiple pathogens or biomarkers within a single sample, providing a comprehensive diagnostic profile and conserving resources [24]. The ability of split Cas12a, especially variants like MeCas12a, to distinguish between single nucleotide polymorphisms (SNPs) with high fidelity is critical for applications such as:

Pathogen Variant Identification: Differentiating between closely related viral strains or bacterial species is crucial for epidemiological tracking and guiding treatment strategies [24].

Genotyping: Identifying genetic mutations or variations associated with disease susceptibility, drug resistance, or personalized medicine [24].

Biomarker Panels: Simultaneously detecting multiple disease biomarkers from a single blood or saliva sample to improve diagnostic accuracy and provide a more complete clinical picture [31]. The modular nature of split Cas12a components lends itself to complex assay design, enabling the development of sophisticated multiplexed formats that require high specificity and minimal cross-reactivity between different targets [32]. This capability significantly enhances the utility of Cas12a in diverse clinical diagnostic scenarios, from infectious diseases to genetic screening [32].

### 4.6. Performance Metrics of Split CRISPR/Cas12a Systems

The advent of CRISPR/Cas systems has revolutionized molecular diagnostics, offering powerful tools with remarkable specificity and versatility [40]. Among these, split CRISPR/Cas12a technology has emerged as a particularly promising avenue, enhancing the practicality of diagnostic assays in diverse real-world contexts, particularly within clinical settings [41,42]. The modular design and conditional activation mechanisms of split systems enable highly sensitive, specific, and rapid detection of a wide array of targets, from nucleic acids to proteins, making them ideal for point-of-care (POC) applications. The performance of split CRISPR/Cas12a systems is defined by key metrics: sensitivity, specificity, and detection speed [15,42]. These systems demonstrate impressive sensitivity, with limits of detection (LoD) often reaching attomolar or femtomolar concentrations, even without pre-amplification steps. For instance, a Framework-Hotspot reporter (FHR) enhanced CRISPR-Cas12a platform achieved sensitive detection down to 100 fM for pathogen nucleic acids and tumor protein markers, with results available in under 15 min [15,42,43]. Another split Cas12a system (SCas12a) demonstrated an LoD of 10 fM for miRNA detection, again without the need for reverse transcription or pre-amplification. When combined with amplification techniques like recombinase polymerase amplification (RPA), sensitivity can be further boosted, allowing detection of targets like human papillomavirus (HPV) at attomolar concentrations [15]. Specificity is another hallmark of split CRISPR/Cas12a systems. The precise coordination required for the reassembly of split components ensures minimal off-target activity, critical for accurate diagnostics [15]. These systems can effectively discriminate between highly similar sequences, including distinguishing mature miRNAs from their precursors and identifying single-nucleotide polymorphisms (SNPs) [44]. For example, the SCas12aV2 assay, an enhanced split Cas12a system, exhibits high specificity for SNPs and successfully identifies SARS-CoV-2 infections [44]. Similarly, a recent RPA-CRISPR/Cas12a-LFS method designed for Duck circovirus (DuCV) detection showed 100% consistency with real-time quantitative polymerase chain reaction (qPCR) and no cross-reactivity with six other avian viruses, confirming its high specificity [45]. Speed is paramount in clinical diagnostics, and split CRISPR/Cas12a systems offer rapid turnaround times. Many platforms deliver results within minutes to an hour. The RPA-CRISPR/Cas12a-LFS system for DuCV detection yields results in just 45 min [45]. Another method for miRNA detection, based on a split-T7 switch, can achieve femtomolar detection within 1 h. Moreover, Cas12a-Graphene Field-Effect Transistor (GFET) devices can differentiate positive cases from serum samples within 5 min [46]. The robust performance of split CRISPR/Cas12a technology translates directly into practical applications for clinical sample analysis. These systems have been successfully validated for detecting various biomarkers and pathogens across a spectrum of human and animal samples, including blood, serum, saliva, urine, nasopharyngeal swabs, and tracheal aspirates [47,48,49]. The ability to perform sensitive and specific detection directly from complex matrices, often without extensive pre-processing or amplification, significantly enhances their utility in point-of-care settings, reducing the need for expensive equipment and specialized personnel [46].

This Table 2 illustrates the diverse applications and robust performance of split CRISPR/Cas12a systems in clinical sample diagnostics. They offer high sensitivity crucial for early disease detection, exceptional specificity to prevent false positives, and rapid detection times, which are vital for timely intervention [46]. The flexibility in sample types, ranging from easily accessible saliva and urine to more complex blood and tissue aspirates, further underscores their practical applicability in various diagnostic settings. Such attributes make split CRISPR/Cas12a a formidable tool, addressing the pressing need for rapid, sensitive, and user-friendly diagnostic platforms in modern healthcare. The development of split CRISPR/Cas12a technology represents a significant stride in molecular diagnostics. Its inherent advantages—high sensitivity, robust specificity, and swift detection capabilities—make it profoundly practical for widespread clinical adoption. The successful application across a multitude of clinical sample types, often without complex pre-amplification steps, positions these systems as critical components for next-generation point-of-care testing and accessible precision medicine. As research continues to refine these systems, their role in disease surveillance, early diagnosis, and personalized treatment strategies is poised to expand even further.

## 5. Advancements and Challenges in Enhancing Split CRISPR/Cas12a Systems for Molecular Diagnostics

The split CRISPR/Cas12a system has revolutionized molecular diagnostics, but issues like structured RNA detection and multiplexing scalability challenge its efficacy. This subsection explores current innovations addressing these limitations, highlighting advancements in design, stability, and activity regulation to enhance performance and expand clinical applicability of these technologies (Table 3).

### 5.1. Detection of Highly Structured RNA

Current iterations of split CRISPR/Cas12a systems exhibit diminished efficiency and sensitivity when addressing RNA with intricate secondary structures, which poses challenges in clinical contexts. The goal is to develop and validate universal scaffold and activator designs tailored for various structured RNA targets, while integrating machine learning approaches to predict optimal split crRNA configurations. Despite advancements like SCas12aV2, structured RNA detection remains a bottleneck, necessitating solutions to overcome steric hindrance for broader applicability in clinical diagnostics 9/9/202552.

### 5.2. Multiplexing Scalability and Cross-Reactivity

The multiplexed detection capabilities of pooled split crRNAs face notable challenges concerning signal interference and cross-reactivity, which impede the scalability of assays. Solutions include the design of orthogonal split crRNA sets that exhibit minimal cross-talk, paired with the development of computational tools for the efficient design of multiplex assays, followed by experimental validation in complex biological samples. While multiplexing is pivotal for cost-effective diagnostics, precise design is essential to minimize false positives and maintain assay sensitivity [52,53].

### 5.3. Amplification-Free Sensitivity in Complex Matrices

Split CRISPR/Cas12a assays devoid of amplification often reveal reduced sensitivity when applied to clinical or environmental samples due to matrix effects. Approaches to address this issue involve engineering split crRNA and Cas12a variants that exhibit enhanced stability and activity in complex matrices, alongside the development of sample preparation methods that align with amplification-free detection protocols. Given the necessity for high sensitivity without amplification in real-world scenarios, this area remains underexplored [54,55].

### 5.4. Standardization of Split crRNA Assembly and Stability

Variability in the efficiency and stability of split crRNA-Cas12a complex reassembly across varied conditions undermines assay reproducibility. A systematic characterization of split crRNA assembly kinetics and stability under different environmental factors—such as temperature, ionic strength, and biological fluids—is essential. Establishing standardized protocols and reagents will form the foundation for reliable reassembly, a critical aspect that currently lacks comprehensive characterization [56].

### 5.5. Integration of Activity Regulation Mechanisms

Existing methods for activity control (e.g., RNA G-quadruplex, elongation-caged activators) introduce complexity and require optimization, thereby limiting practical deployment. The development of simplified, modular activity regulation strategies, compatible with split crRNA frameworks, is critical. Exploring light- or small molecule-induced switches for on-demand activation could enhance the specificity of Cas12a activity while simplifying assay design and operation [53,57,58].

### 5.6. Expansion to Non-Nucleic Acid Targets with Split Systems

While aptamer-based CRISPR/Cas12a assays demonstrate the capability to detect proteins and small molecules, their integration with split crRNA technology remains limited. Efforts should focus on engineering split crRNA-Cas12a platforms that incorporate aptamer or allosteric ribozyme modules, allowing for multiplexed detection of non-nucleic acid targets, with validation in clinical samples. Expanding the detection repertoire to non-nucleic acid targets enhances the diagnostic scope, but compatibility issues with split systems still need to be resolved [59,60,61].

### 5.7. Real-Time and In Situ Detection Capabilities

Few studies have successfully leveraged split CRISPR/Cas12a systems for real-time or intracellular detection with low background noise. The advancement of split crRNA designs and delivery methodologies specifically for live-cell imaging and real-time monitoring is vital, alongside optimizing activation strategies that minimize background interference. Enhancing intracellular and dynamic detection will expand the applications of these systems but necessitates refined signal control and delivery methods [62,63].

### 5.8. Universal Design Frameworks for Split crRNA

The absence of generalized design rules for split crRNA sequences and activators hinders the efficient development of assays for novel targets. To address this, the creation of computational platforms that integrate both structural and biochemical data for predicting effective split crRNA designs is imperative; considerations for target secondary structure should also be incorporated. Streamlining the design process can facilitate faster assay development and reduce the trial-and-error phase [52,54].

### 5.9. Long-Term Stability and Storage of Split Components

The stability of split crRNA and Cas12a complexes during storage and transport has not been thoroughly investigated, which hampers their deployment in the field. Research focused on lyophilization and stabilization formulations for these components is necessary, along with assessments of activity retention over time under varying conditions. Ensuring stability is crucial for point-of-care applications and resource-limited settings, yet available data in this area remain sparse [64].

### 5.10. Comprehensive Clinical Validation

Most split CRISPR/Cas12a assays lack extensive validation across diverse clinical cohorts and sample types. Conducting large-scale clinical studies that compare the performance of split CRISPR/Cas12a assays against gold-standard diagnostic methods across various diseases and sample matrices will be essential. Comprehensive clinical validation is vital for transitioning promising assays into practical diagnostic tools [53,54].

**Table 3:** Challenges and Solutions in Advancing Split CRISPR/Cas12a Systems for Diagnostic Applications.

Challenging Area	Limitations	Potential Solutions	Insights
Detection of Highly Structured RNA	Current split CRISPR/Cas12a systems show reduced efficiency and sensitivity when detecting RNA with complex secondary structures, limiting clinical applicability.	Develop and validate universal scaffold and activator designs optimized for diverse structured RNA targets; integrate machine learning to predict optimal split crRNA configurations for structured RNAs.	Structured RNA detection remains a bottleneck despite advances like SCas12aV2; overcoming steric hindrance is critical for broad clinical diagnostics [54].
Multiplexing Scalability and Cross-Reactivity	Multiplexed detection using pooled split crRNAs faces challenges in signal interference and cross-reactivity, limiting assay scalability.	Design orthogonal split crRNA sets with minimal cross-talk; develop computational tools for multiplex assay design; experimentally validate multiplexing in complex biological samples.	Multiplexing is essential for cost-effective diagnostics but requires precise design to avoid false positives and maintain sensitivity [52,53].
Amplification-Free Sensitivity in Complex Matrices	Amplification-free split CRISPR/Cas12a assays often show decreased sensitivity in clinical or environmental samples due to matrix effects.	Engineer split crRNA and Cas12a variants with enhanced stability and activity in complex matrices; develop sample preparation methods compatible with amplification-free detection.	Maintaining high sensitivity without amplification in real-world samples is necessary for point-of-care use but remains underexplored [54,55].
Standardization of Split crRNA Assembly and Stability	Variability in reassembly efficiency and stability of split crRNA-Cas12a complexes under different conditions affects assay reproducibility.	Systematically characterize split crRNA assembly kinetics and stability across temperature, ionic strength, and biological fluids; develop standardized protocols and reagents.	Reliable reassembly is foundational for assay consistency but lacks comprehensive characterization [56].
Integration of Activity Regulation Mechanisms	Current activity control methods (e.g., RNA G-quadruplex, elongation-caged activators) add complexity and require optimization, limiting practical deployment.	Develop simplified, modular activity regulation strategies compatible with split crRNA systems; explore light- or small molecule-controlled switches for on-demand activation.	Precise Cas12a activity control improves specificity but often complicates assay design and operation [53,57,58].
Expansion to Non-Nucleic Acid Targets with Split Systems	While aptamer-based CRISPR/Cas12a assays detect proteins and small molecules, integration with split crRNA technology is limited.	Engineer split crRNA-Cas12a platforms coupled with aptamer or allosteric ribozyme modules for multiplexed detection of non-nucleic acid targets; validate in clinical samples.	Non-nucleic acid detection broadens diagnostic scope, but split system compatibility remains underdeveloped [59,60].
Real-Time and In Situ Detection Capabilities	Few studies demonstrate split CRISPR/Cas12a systems for real-time or intracellular detection with minimal background.	Develop split crRNA designs and delivery methods for live-cell imaging and real-time monitoring; optimize low-background activation strategies.	Intracellular and dynamic detection expands applications but requires improved signal control and delivery [62,63].
Universal Design Frameworks for Split crRNA	Lack of generalized design rules for split crRNA sequences and activators hinders rapid assay development for new targets.	Create computational platforms integrating structural and biochemical data to predict effective split crRNA designs; incorporate target secondary structure considerations.	Streamlined design accelerates assay development and reduces trial-and-error [5].
Long-Term Stability and Storage of Split Components	Stability of split crRNA and Cas12a complexes during storage and transport is insufficiently studied, impacting field deployment.	Investigate lyophilization and stabilization formulations for split crRNA and Cas12a; assess activity retention over time under varied conditions.	Stability is critical for point-of-care and resource-limited settings but data are scarce [64].
Comprehensive Clinical Validation	Most split CRISPR/Cas12a assays lack extensive validation in diverse clinical cohorts and sample types.	Conduct large-scale clinical studies comparing split CRISPR/Cas12a assays with gold-standard diagnostics across diseases and sample matrices.	Clinical validation is essential to translate promising assays into practical diagnostics [53,54].

## 6. Future Perspectives and Outlook

Looking ahead, the trajectory of split CRISPR/Cas12a technology points toward even greater modularity, universality, and real-world adaptability. Key future directions include:

- Universal Split Reagent Toolkits: Ongoing efforts are focused on producing standardized, ready-to-use libraries of split crRNAs, activators, and scaffolds for rapid plug-and-play adaptation to any new target, supporting decentralized and field-based diagnostics [15].
- Advanced Multiplexed and Logic-Gated Diagnostics: The sophistication of logic operations and barcode-based multiplexing is expected to increase, enabling concurrent testing for numerous pathogens or gene variants in a single workflow—with built-in logic controls for decision-making based on multi-analyte status [65,66].
- Sample Compatibility and Automation: Strategies for robust direct-sample-to-answer workflows—overcoming inhibitory effects, leveraging low-cost extraction-free chemistries, and further automating the detection process with microfluidics and smartphone integration—are advancing point-of-care and at-home testing applications [67,68].
- Integration with Other Sensing Modalities: Hybridization with electrochemical, optical, and nanoparticle-based readouts, as well as integration with DNA nanotechnology for enhanced signal generation and background suppression, will continue to push sensitivity and operational flexibility [69,70].
- Synthetic Biology and Therapeutic Applications: The use of programmable proximity-activated or chemically gated split components is anticipated not just in diagnostics but as control modules for in vivo genetic circuits, regulated gene therapy, and responsive drug delivery systems [71,72].
- Improved Analytical Performance: Rational optimization—such as iterative crRNA design and the use of redundant, modular interfaces—will further reduce detection limits and shorten time-to-result for ultra-rapid, trace-level sensing, including single-cell and environmental pathogen monitoring [73].

Recent advancements in split CRISPR/Cas12a sensor technology have underscored a revolution in programmable, highly sensitive, and versatile biosensing platforms [15]. With strategic engineering innovations, increasing multiplexing capacity, and creative integration with both emerging nanotechnologies and consumer electronics, split technology is poised to fundamentally transform the landscape of molecular diagnostics, environmental monitoring, and personalized medicine in the years ahead [1,8,74]. Ongoing research addressing the current limitations—in activity, multiplexing, and real-world robustness—will be essential for widespread adoption. However, the modular nature and inherent programmability of split CRISPR/Cas12a sensors provide a broad foundation for future innovation and applications [6,74].

## 7. Conclusions

Split CRISPR/Cas12a-based sensors represent a major advancement in molecular diagnostics, offering a modular, programmable alternative to traditional CRISPR systems with enhanced specificity, reduced background noise, and flexible assay design. By separating key components such as crRNA or target activators, these systems enable conditional activation only in the presence of specific targets, making them highly adaptable for applications like infectious disease detection, environmental monitoring, and personalized medicine. Despite current challenges—including reduced activity, slower kinetics, and interference from complex biological samples—ongoing innovations in molecular engineering, chemical activation, and nanotechnology integration are steadily overcoming these limitations. These advancements are paving the way for simplified, one-pot diagnostic platforms that are robust, sensitive, and easy to deploy in real-world settings. As research continues to expand target versatility, improve multiplexing capabilities, and enhance performance under diverse conditions, split CRISPR/Cas12a technology is poised to become a cornerstone of next-generation biosensing and diagnostic tools, with the potential to significantly shape the future of precision medicine and public health.

## Figures and Tables

**Table 1:** Difference between Complete Cas12a System and Split Cas12a System.

Parameter	Complete Cas12a System	Split Cas12a System
Molecular Design	A single, full-length Cas12a effector protein containing the RuvC domain. It is guided by a single crRNA, which forms an R-loop with the target DNA. The enzyme itself performs both target recognition and collateral cleavage [21,24].	The Cas12a protein is engineered into two or more inactive fragments. These fragments must reassemble into a functional complex upon interaction with the target nucleic acid or under specific engineered conditions [22,24].
Sensitivity	High sensitivity, typically reaching picomolar detection limits without pre-amplification, and femtomolar range with engineered crRNAs. Can detect as low as 50 RNA copies per µL when combined with isothermal amplification techniques like RT-LAMP. Clinical samples for SARS-CoV-2 detection show high sensitivity, even for samples requiring 30–39 RT-qPCR threshold cycles. Manganese (Mn)-enhanced Cas12a systems (MeCas12a) can detect as low as five copies of target RNA. Nucleic acid amplification-free fluorescent biosensors can detect targets like breast cancer gene-1 (BRCA-1) with very high sensitivity in 30 min [19,20,23,24,25].	Very high sensitivity is maintained, with manganese enhancement enabling detection of target RNAs as low as five copies. While reassembly kinetics might introduce minor kinetic delays, overall sensitivity remains comparable to complete systems, especially when coupled with pre-amplification. The controlled activation minimizes background noise, effectively enhancing the signal-to-noise ratio and thus contributing to robust low-copy detection [24].
Specificity	High specificity through sequence-specific recognition by the crRNA-Cas12a complex. However, studies indicate that Cas12a can exhibit pervasive sequence-specific nicking of randomized target libraries, tolerating up to four mismatches in the DNA-RNA hybrid sequences, and robust non-specific nicking of dsDNA when activated by a target. This can lead to some background activity or potential off-target effects depending on the ortholog and crRNA sequence. In specific assays, 100% clinical specificity with no false positives for SARS-CoV-2 detection has been reported [20,23,25].	Enhanced specificity is a key advantage, as functional cleavage activity only occurs upon target-induced reassembly of the Cas12a fragments. This conditional activation significantly reduces background (off-target) cleavage and minimizes false positives. MeCas12a, for instance, is highly specific and can distinguish between single-nucleotide polymorphisms (SNPs) differing by only a single nucleotide. This makes it particularly valuable for applications requiring precise discrimination [22,24].
Detection Time	Rapid detection times, typically within 30 to 60 min when integrated with amplification methods. Single-tube assays for SARS-CoV-2 detection combining RT-LAMP and Cas12a can yield results within 40 min. Integration with recombinase-aided amplification (RAA) can allow detection within 1.5 h in automated point-of-care devices. Cas12a-based nucleic acid amplification-free biosensors can detect targets in 30 min [23,25].	Detection times generally fall within minutes to an hour. The reassembly kinetics of the split fragments might introduce a slight delay compared to the direct activation of a complete system. However, advancements like manganese-enhanced systems can accelerate the collateral cleavage, maintaining rapid diagnostics. For instance, a manganese-enhanced Cas12a system (MeCas12a) demonstrates rapid collateral cleavage kinetics [22].
Operational Requirements	Simpler reagent composition involving a single, full-length Cas12a protein, a crRNA, and reporter molecules. Reaction conditions are generally straightforward, often compatible with isothermal amplification temperatures (e.g., 62 °C for RT-LAMP). Integration into automated microfluidic platforms is feasible. Optimized crRNA length and modifications (e.g., 7-mer DNA extension) can universally enhance sensitivity and specificity [23,25].	More complex reagent composition potentially requiring optimization for stable reassembly of the split protein fragments. This may necessitate specific buffer compositions, temperature ranges, or other conditions to ensure efficient fragment association. Guide RNA folding and its interaction with the direct repeat are crucial for Cas12a activity, which is also relevant for split systems [22,24].
Stability and Storage	Generally robust under appropriate storage conditions for enzyme stability. The complete enzyme form typically maintains its activity without specific reassembly challenges. However, its inherent moderate background activity persists during storage and operation. Improved Cas12a variants with enhanced activity and altered PAM specificities are continually being developed [23,24,26].	Reagents are generally robust under suitable storage conditions. The controlled activation mechanism through assembly can potentially lead to improved operational stability during assays by minimizing nonspecific activity prior to target binding. Specific data on shelf life differences between split and complete systems under various conditions are not explicitly detailed in the provided documents, but the engineered nature suggests careful characterization for long-term stability may be required [24].
Scalability and Adaptability	Highly adaptable for integration into automated and high-throughput platforms. Automated systems like CASMEAN can detect nucleic acids rapidly, integrating recombinase-aided amplification and Cas12a detection within 1.5 h, making them suitable for point-of-care (POC) applications. Single-tube assays are beneficial for future POC applications and can be read by smartphone cameras. Can be scaled for large-scale wastewater surveillance to detect SARS-CoV-2, processing over 100 samples a day [23,27,28].	Adaptable for multiplexed detection, particularly benefiting from enhanced specificity for discriminating closely related sequences like SNPs. This high discrimination is valuable in complex samples or when targeting multiple biomarkers simultaneously. Maintains high sensitivity when coupled with amplification, suitable for detecting very low copy numbers rapidly in multiplexed settings. The modularity of split components could potentially facilitate future engineering for complex biosensing applications.
Key Applications	Genome editing (e.g., HIV inactivation, plant genome engineering). Rapid, high-throughput diagnostics for infectious diseases (e.g., SARS-CoV-2, Pseudomonas aeruginosa). Amplification-free biosensing for cfDNA [23,26,27].	High-accuracy diagnostics, particularly where distinguishing closely related targets or reducing false positives is critical (e.g., SNP discrimination, viral detection). Research tool for controlled biological studies and potential therapeutic applications requiring conditional gene editing.

**Table 2:** Comparative Analytical Performance of Split CRISPR/Cas12a Systems in Clinical Applications.

System Name	Detection Time	Sample Type	Sensitivity (LoD)	Specificity Level	Reference
FHR-enhanced Cas12a	<15 min	Clinical samples (protein markers, pathogen nucleic acids)	100 fM	Not explicitly stated	[42]
SCas12a (miRNA)	Fast	Patient samples (HPV DNA)	10 fM (miRNA); attomolar (HPV DNA)	Distinguishes mature/pre-miRNA, outperforms wild-type Cas12a for DNA point mutations	[15]
RPA-CRISPR/Cas12a-LFS (DuCV)	45 min	Waterfowl samples (clinical)	2.6 gene copies	100% consistency with qPCR, no cross-reactivity with 6 other avian viruses	[45]
Cas12a-GFET (Mycobacterium tuberculosis)	5 min	Serum samples	2.42 × 10^18^ M	Differentiates positive cases	[43]
One-pot method (general)	<60 min	Not explicitly stated (implied clinical applicability)	Attomolar (DNA, RNA)	Not explicitly stated	[8,41]
sPAM-enhanced Cas12a (miR-183)	25 min	Clinical serum samples	0.40 aM	Exceptional for miR-183 from other miRNAs	[50]
Cas12a (SARS-CoV-2)	Not explicitly stated	Nasopharyngeal swabs, saliva, tracheal aspirates	Not explicitly stated	Discriminatory for SARS-CoV-2 and B.1.1.7 lineage	[47]
SCas12aV2 (SARS-CoV-2, bacteria)	Not explicitly stated	Clinical samples	246 aM (pooled activators); 10 pM (single-site)	High specificity for SNPs	[44]
HCR-CRISPR/Cas12a (α-synuclein)	Not explicitly stated	Human serum samples	9.33 pM	Satisfactory applicability	
RPA-CRISPR/Cas12a (SFTSV)	40 min	Clinical samples	3 copies (L gene)	No cross-reactivity with other RNA viruses, 100% agreement with Q-PCR	[40]
Split-T7 switch Cas12a (miR-21)	<1 h	Cell lines (miR-21)	Femtomolar	Not explicitly stated	
Electrochemical biosensor (miRNA-155, ctDNA)	2.6 h	Human serum, cancer cell lysates	aM levels	Outstanding reliability and accuracy	[46]
FCAS-CRISPR/Cas12a (miRNA-10b)	Not explicitly stated	Clinical serum samples (glioma patients)	5.53 fM	Effective recognition of tumor cells	[51]

## Data Availability

Not applicable.
